# Allostatic load-cardiovascular disease associations and the mediating effect of inflammatory factors: a prospective cohort study

**DOI:** 10.3389/fcvm.2025.1724572

**Published:** 2026-01-05

**Authors:** Shuai Xu, Ge Zhang, Yudi Xu, Chaoyang Yu, Ruhao Wu, Zhengrui Li, Teng Li, Xinyue Cui, Xufeng Huang, Shujing Zhou, Yahui Han, Haonan Zhang, Shiqian Zhang, Yufeng Jiang

**Affiliations:** 1 Department of Cardiology, The Fourth Affiliated Hospital of Soochow University, Suzhou Dushu Lake Hospital, Medical Center of Soochow University, Suzhou, China; 2 Institute for Hypertension, Soochow University, Suzhou, China; 3 Department of Cardiology, The First Affiliated Hospital of Zhengzhou University, Zhengzhou, Henan, China; 4 Department of Neurology, The First Affiliated Hospital of Zhengzhou University, Zhengzhou, Henan, China; 5 Department of Vascular Surgery, First Hospital of Tsinghua University, Beijing, China; 6 Department of Respiratory and Critical Care Medicine, The First Affiliated Hospital of Zhengzhou University, Zhengzhou, Henan, China; 7 Department of Oral and Maxillofacial Head and Neck Oncology, Ninth People Hospital, Shanghai Jiao Tong University School of Medicine, Shanghai, China; 8 Faculty of Medicine, University of Debrecen, Debrecen, Hungary; 9 School of Pharmacy, Harbin Medical University School of Pharmacy, Harbin, China

**Keywords:** allostatic load, cardiovascular disease, UK Biobank, inflammatory factors, prospective

## Abstract

**Background:**

Allostatic load (AL) captures multisystem dysregulation that accrues with chronic stress and may shape cardiovascular disease (CVD) risk through neuroendocrine and immune pathways. Robust population-scale evidence clarifying the exposure-response pattern and the extent of inflammatory mediation remains limited.

**Methods:**

In the UK Biobank, we analyzed 205,504 adults free of CVD at baseline from 502,366 recruited. An AL score was assembled from 12 routinely measured biomarkers. Incident CVD was ascertained via linkage to hospital and mortality records. We estimated adjusted hazard ratios (HRs) using Cox models and assessed nonlinearity with restricted cubic splines; robustness was evaluated in prespecified subgroups and sensitivity analyses. We also investigated the role of the mediating effect of inflammatory factors in the AL-CVD relationship.

**Results:**

Higher AL tracked with progressively greater CVD risk in a graded, non-linear pattern. Relative to AL = 0, AL> = 6 was associated with HR 2.15 (95% CI 1.99–2.33). Eight inflammatory markers met retention criteria for mediation; neutrophil count mediated 4.73% of the AL-CVD association. Group contrasts across AL tertiles indicated Cohen's d near 0.5 for several markers, largest for no-AL vs. high-AL.

**Conclusion:**

Elevated AL is linked to higher incident CVD with a non-linear exposure-response, and neutrophil-centric inflammation accounts for a measurable portion of the association. These findings support integrating stress-biology constructs and inflammatory profiling into cardiovascular risk assessment and prevention frameworks.

## Introduction

Cardiovascular disease (CVD) is a group of conditions that affect the heart and blood vessels, including heart attack and stroke (1). CVD remain the predominant cause of adult mortality worldwide (2), underscoring the need to refine risk assessment beyond conventional factors. Stress refers to environmental or psychosocial challenges that elicit adaptive physiological responses. Allostatic load (AL) denotes the cumulative, multisystem biological cost of repeated or chronic stress exposure, arising from sustained activation of the hypothalamic-pituitary-adrenal axis and sympathetic nervous system. These neuroendocrine changes promote leukocyte demargination with a neutrophil-predominant profile, oxidative stress, endothelial dysfunction, and pro-atherogenic signaling-plausible pathways by which elevated AL may increase CVD risk.

The principal behavioural drivers of CVD are poor diet, inactivity, tobacco use, and hazardous alcohol intake; these behaviours manifest clinically as raised blood pressure, abnormal glucose and lipid profiles, and overweight/obesity. Prior studies suggest that higher AL relates to adverse cardiometabolic outcomes, but critical gaps persist regarding the shape of the AL-CVD exposure-response in the general population and the contribution of inflammatory pathways (3–5). Interest is growing in AL as a composite index of chronic stress, designed to capture the cumulative impact of sustained stressors and life event (6, 7). AL, as a composite indicator, involves the dysregulation of multiple physiological systems (8). It is hypothesised that chronic stress leads to an overreaction of the immune system manifested as a chronic inflammatory response. Persistent low-grade inflammation may drive the development of several chronic conditions-cardiovascular disease, diabetes, and cancer among them (9, 10). A previous Chinese study reported that high baseline AL was associated with an increased risk of incident CVD and all-cause mortality (11). Despite suggestive evidence, the relationship between AL and incident cardiovascular disease-and the contribution of inflammatory pathways-remains incompletely defined. Using the UK Biobank, we examined how AL relates to new-onset CVD, characterized the exposure-response with restricted cubic splines, and quantified mediation by leukocyte-derived and composite inflammatory indices.

Herein, we explored the relationship between AL and CVD and whether inflammatory markers served as mediators in their relationship dependent on UK Biobank database.

## Methods

### Research design

UK Biobank is a large, population-based resource recruiting middle-aged adults across the United Kingdom with harmonized questionnaires, standardized physical measurements, biospecimen collection, and prospective linkage to hospital admissions and mortality registries (12). The dataset was the UK Biobank cohort comprising 502,366 individuals. For this analysis, we excluded participants with missing AL components (*n* = 135,124), evidence of CVD at baseline (*n* = 16,449), or missing prespecified covariates (*n* = 145,289), yielding 205,504 individuals free of CVD at baseline. Follow-up extended from baseline assessment to the first CVD event, death, loss to follow-up, or end of linkage.

### Ethics approval and consent

The UK Biobank received ethical approval from the North West Multi-centre Research Ethics Committee (REC 11/NW/03820). All participants provided written informed consent. Use of the resource for this study was approved under UK Biobank application 68136.

### Incident CVD ascertainment

The onset of CVD in the UK Biobank was identified through hospitalisation records and death registrations. CVD was identified according to the International Classification of Diseases (ICD). ICD-10 codes I20, I21, I22, I23, I24, I25, I60, I61, I62, I63, I64 and I69 were used to identify CVD (13).

### Assessment of allostatic load

Guided by prior literature and UK Biobank variable availability, we constructed an allostatic load (AL) score from 12 routinely measured baseline biomarkers (14): glycated haemoglobin (HbA1c), high-density lipoprotein cholesterol (HDL), low-density lipoprotein cholesterol (LDL), total cholesterol (TC), triglycerides (TG), waist-hip ratio (WHR), systolic and diastolic blood pressure (SBP and DBP, respectively), pulse rate (PR), C-reactive protein (CRP), insulin-like growth factor-1 (IGF-1) and creatinine (Cre). The collection of these biomarkers occurred at the baseline of the study and subsequently for the purpose of analysis following the diagnosis of CVD. For HbA1c, LDL, TC, TG, WHR, SBP, DBP, PR, CRP, Cre, greater than or equal to the upper quartile of the population (75th percentile) is defined as a score of 1, otherwise 0. For HDL, IGF-1, less than or equal to the lower quartile of the population (25th percentile) is defined as a score of 1, otherwise 0 points. The final score is the sum of the 12 scores, ranging from 0 to 12 (cutoff values for each variable are in Supplementary Table S1) (15, 16). We parameterized AL in complementary ways: (i) seven-level categories (0, 1, 2, 3, 4, 5, ≥6) to preserve the discrete, integer nature of the score and to display a fine-grained dose–response, and (ii) tertiles to facilitate clinical interpretability, enable parsimonious subgroup reporting, and reduce sparse cells. We additionally modeled AL as continuous with restricted cubic splines to check functional form (14).

### Covariates

In this study, covariates included socio-demographic characteristics (age, race, sex, employment, education, deprivation, income), lifestyle factors (MET group, alcohol_status, BMI + smoking_status) and baseline medication use (medication_cholesterol, medication BP, medication insulin). Age (continuous) was determined by baseline or date of birth; race (Asian, black, white, mixed and other), sex (male or female) and employment status (employed or unemployed) were self-reported at baseline. Educational attainment (graduate vs. non-graduate) was dichotomised based on the highest level of formal education received. The Townsend Poverty Index (continuous), constructed on the basis of the postcode of residence, was used to assess socioeconomic deprivation, with higher scores indicating poorer socioeconomic status (17). Annual household income (less than 18,000, 18,000–100,000 and more than 100,000), smoking (current, ever, never) and alcohol consumption [never, occasional, frequent (daily or almost daily)] were obtained from the baseline questionnaire. Body mass index (continuous) was measured as weight (kilograms)/height (square metres) based on baseline anthropometric data. Receipt of insulin therapy (yes or no), use of antihypertensive medication (yes or no) and use of cholesterol-lowering medication (yes or no) were obtained from the baseline questionnaire.

### Statistical analysis

The data on baseline characteristics are represented as mean ± standard deviation of age, BMI and deprivation, and number and frequency of other categorical variables. The analysis of categorical and continuous variables between the non-CVD and CVD groups was conducted using chi-square tests and Student's *t*-tests, respectively. The association of AL and its associated covariates with the risk of developing CVD was assessed using a Cox proportional risk regression model. Hazard ratios (HRs) and 95% confidence intervals (CIs) are reported grouped by AL score, using a score of zero as the reference. Time-to-event analyses used Cox models with time since baseline as the time scale. The AL-CVD association was examined both by categories and as a continuous exposure using restricted cubic splines, providing complementary parameterizations that reduce sensitivity to modeling assumptions, including proportional hazards.

In the pursuit of elucidating the potential impact of covariates on the risk of AL and CVD, a series of subgroup analyses were conducted, with the study population stratified by age (less than 65 years young adult; or more than 65 years old adult), BMI (less than 25 non-obesity; or more than 25 as obesity) and other relevant covariates. The degree of interaction between the covariates and AL was assessed by means of likelihood tests. In order to test the robustness of the model, sensitivity analyses were conducted. In addition, reverse causality was ruled out by excluding participants who had experienced an outcome event within three years of the baseline.

We further performed mediation analyses to explore whether AL plays a role in CVD through inflammatory factor mediation. In brief, regression models were constructed with AL as the exposure variable, inflammatory factors as the outcome variable, AL and inflammatory factors as the exposure variable, and CVD as the outcome variable, respectively. The processing and statistical analysis of the data was conducted using R software (version 4.3), with statistical significance being indicated by *P-value <* *0.05*.

## Results

### Baseline characteristics

**Figure 1:**
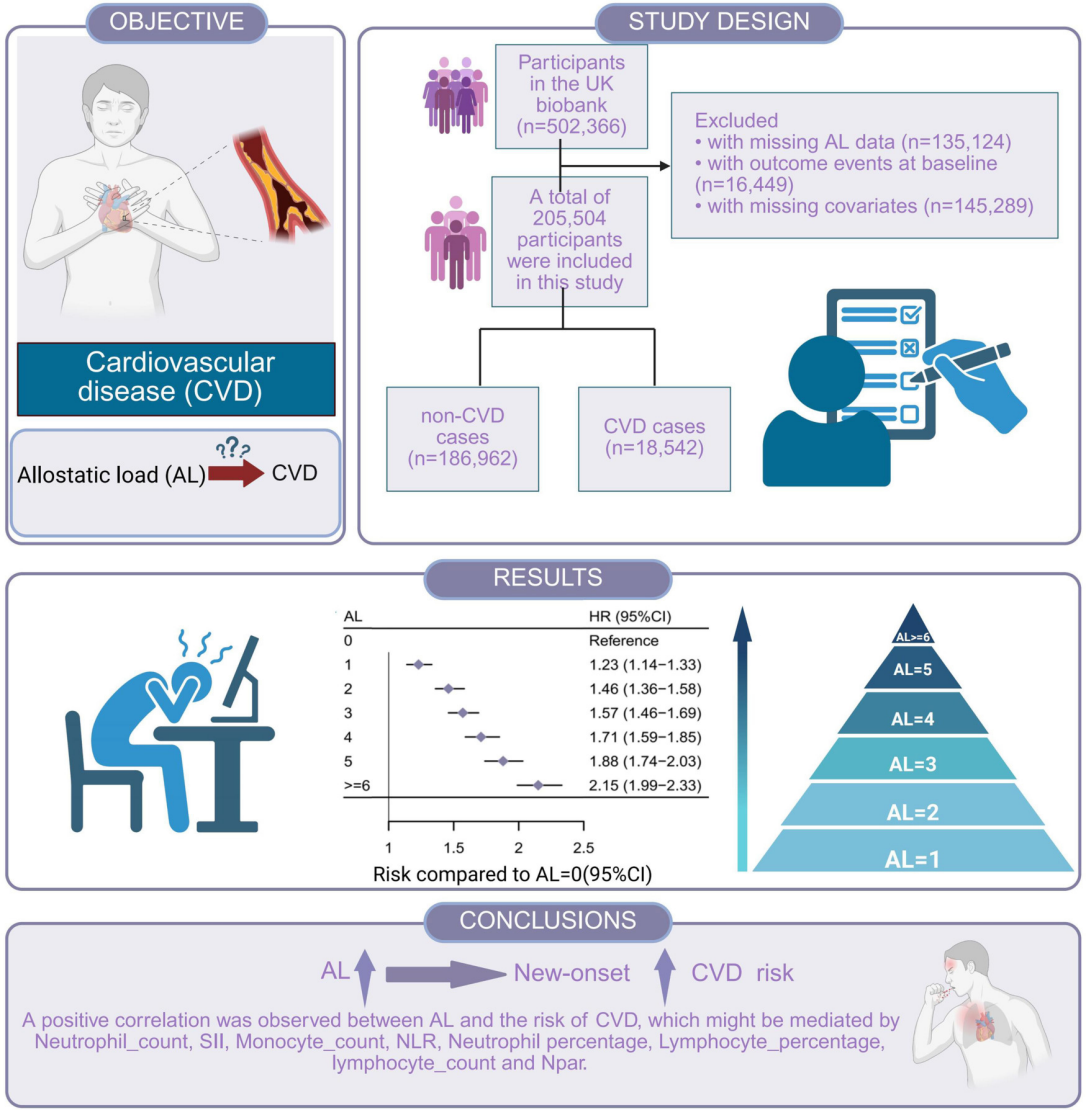
Study flow diagram for participant inclusion/exclusion from UK biobank, showing final analytic cohort *n* = 205,504 (non-CVD *n* = 186,962; incident CVD *n* = 18,542). Elements: source population; exclusions (missing AL *n* = 135,124, baseline CVD *n* = 16,449, missing covariates *n* = 145,289). “Created in BioRender. Zhang, G. (2025) https://BioRender.com/su0o3sk”.

A total of 502,366 participants with baseline AL data were identified in the UK Biobank database, of whom 205,504 were ultimately included following the exclusion of individuals with pre-baseline CVD and the deletion of covariates. We have created a flowchart to illustrate our research (Figure 1). During the 13-year follow-up period, 18,542 individuals were diagnosed with CVD. The baseline characteristics of all individuals included in the study are listed in Table 1. Participants with CVD were found to be more likely to be older, Asian, female, obese, smokers, and individuals with a lower educational attainment, unemployed, and from a poorer socioeconomic status. They were also more likely to have a lower income, a higher MET, and not to be taking lipid-lowering and antihypertensive medications, and not to be using insulin, in comparison to participants without CVD.

**Table 1:** Baseline characteristics of participants by incident CVD status (*n* = 205,504).

Var	level	0	1	*p*
N		1,86,962	18,542	
Age. mean. SD.		54.64 (8.01)	59.33 (7.13)	<0.001
Race…	Asian	3,283 (1.76)	456 (2.46)	<0.001
X	Black	2,307 (1.23)	164 (0.88)	
X.1	Mixed and Others	2,559 (1.37)	200 (1.08)	
X.2	White	1,78,813 (95.64)	17,722 (95.58)	
Sex…	Female	99,631 (53.29)	6,243 (33.67)	<0.001
X.3	Male	87,331 (46.71)	12,299 (66.33)	
BMI.mean.SD.		26.93 (4.53)	28.25 (4.77)	<0.001
Smoke_status…	Current	17,006 (9.10)	2,245 (12.11)	<0.001
X.4	Never	1,07,917 (57.72)	8,861 (47.79)	
X.5	Previous	62,039 (33.18)	7,436 (40.10)	
Alcohol_status…	Current	1,76,197 (94.24)	17,202 (92.77)	<0.001
X.6	Never	5,648 (3.02)	654 (3.53)	
X.7	Previous	5,117 (2.74)	686 (3.70)	
Education_2…	<High School	56,471 (30.20)	5,902 (31.83)	<0.001
X.8	>High School	1,04,071 (55.66)	10,291 (55.50)	
X.9	High School	26,420 (14.13)	2,349 (12.67)	
Employment…	Employed	1,27,340 (68.11)	9,541 (51.46)	<0.001
X.10	Unemployed	59,622 (31.89)	9,001 (48.54)	
Deprivation.mean.SD.		15.49 (12.52)	16.29 (13.26)	<0.001
Income…	18,000 to 100,000	1,46,263 (78.23)	13,684 (73.80)	<0.001
X.11	Greater than 100,000	13,531 (7.24)	859 (4.63)	
X.12	Less than 18,000	27,168 (14.53)	3,999 (21.57)	
MET_group…	High	54,761 (29.29)	5,611 (30.26)	<0.001
X.13	Low	33,930 (18.15)	3,681 (19.85)	
X.14	Moderate	98,271 (52.56)	9,250 (49.89)	
Medication_Cholesterol…	No	1,66,417 (89.01)	13,365 (72.08)	<0.001
X.15	Yes	20,545 (10.99)	5,177 (27.92)	
medication_BP…	No	1,60,119 (85.64)	12,578 (67.84)	<0.001
X.16	Yes	26,843 (14.36)	5,964 (32.16)	
Medication_insulin…	No	1,85,791 (99.37)	18,129 (97.77)	<0.001
X.17	Yes	1,171 (0.63)	413 (2.23)	
AL_score…	0	27,258 (14.58)	909 (4.90)	<0.001
X.18	1	33,604 (17.97)	1,892 (10.20)	
X.19	2	34,035 (18.20)	2,822 (15.22)	
X.20	3	30,654 (16.40)	3,205 (17.29)	
X.21	4	24,294 (12.99)	3,146 (16.97	
X.22	5	17,216 (9.21)	2,679 (14.45)	
X.23	6	10,706 (5.73)	1,895 (10.22)	
X.24	7	5,564 (2.98)	1,168 (6.30)	
X.25	8	2,453 (1.31)	533 (2.87)	
X.26	9	882 (0.47)	213 (1.15)	
X.27	10	254 (0.14)	64 (0.35)	
X.28	11	34 (0.02)	13 (0.07)	
X.29	12	8 (0.00)	3 (0.02)	

Summary of demographics, lifestyle factors, clinical measurements, and medication use in the overall cohort and stratified by non-CVD (*n* = 186,962) vs. incident CVD (*n* = 18,542). Values are mean ± SD, median (IQR), or *n* (%).

Values are mean (SD) for continuous variables and *n* (%) for categorical variables. *P* values are from two-sided tests with *α* = 0.05: chi-square tests for categorical variables and two-sample *t*-tests for continuous variables (Welch's correction applied when variances were unequal).

### Associations of AL with CVD

**Figure 2:**
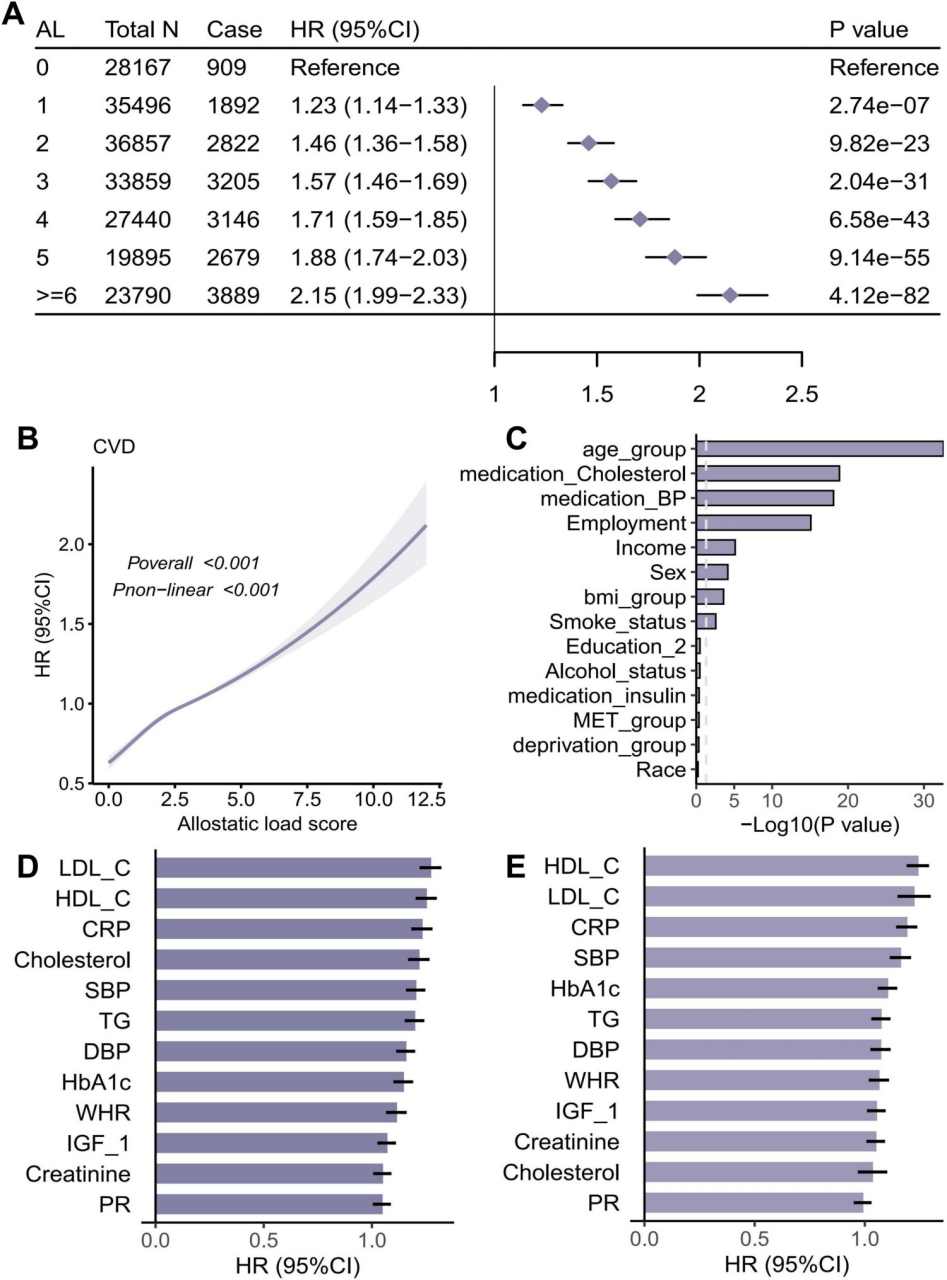
Association between allostatic load (AL) and incident cardiovascular disease (CVD). **(A)** Adjusted hazard ratios by discrete AL score with AL = 0 as reference; **(B)** Restricted cubic spline for AL (knots at standard percentiles) showing non-linear dose-response; **(C)** Prespecified subgroup analyses (age, BMI, sex, race/ethnicity, education, employment, income, deprivation, smoking, alcohol, physical activity, and medication use) with interaction *P* values; **(D,E)** Associations of individual AL components with CVD under sequential adjustments.

The relationship between AL and CVD was examined by developing three COX proportional hazards models. Model 1 was adjusted for age, race, sex, employment, education, deprivation and income. Model 2 was adjusted by adding the MET group, alcohol status, BMI and smoking status to Model 1. Model 3 contains additional adjustments for all covariates in the study. In analyses where AL was modelled as a continuous variable, a positive dose gradient association (*P* < .001) was identified between AL score and CVD risk (see Supplementary Table S2). In model 3, AL scores of 1, 2, 3, 4, 5, and > = 6 had HR (95% CI) of 1.23 (1.14–1.33), 1.46 (1.36–1.58), 1.57 (1.46–1.69), 1.71 (1.59–1.85), 1.88 (1.74–2.03), and 2.15(1.99–2.33), and a significantly increased risk of CVD among participants with an AL score greater than or equal to 6 (HR 2.15, 95% CI 1.99–2.33)(Figure 2A). Restricted cubic splines analysis demonstrated a substantial nonlinear relationship (Pnonlinear < 0.001), thereby signifying a nonlinear correlation between AL and the onset of CVD (Figure 2B). It is noteworthy that following the exclusion of participants who developed CVD within 3 years of the baseline, the results obtained from the sensitivity analyses were analogous to those previously described, thereby indicating the reliability of the study's findings (Supplementary Figure S1).

In subgroup analyses, the covariates were grouped according to their coefficients. The relationship between AL and CVD in each subgroup was explored, and the interaction of AL with each covariate was calculated. It was found that multiple variables interacted significantly with AL (Figure 2C, Supplementary Figure S2, Supplementary Table S3**)**. In a univariate analysis exploring the relationship between individual factors in AL and CVD, Figure 2D demonstrates the relationship between each individual factor (e.g., LDL_C, HDL_C, CRP, etc.) and CVD after correcting for covariates in Model 3. Multiple factors demonstrate HRs greater than 1 and CIs that are narrow and do not span 1, suggesting that these factors significantly increase the risk of CVD. Figure 2E further corrects for the aforementioned covariates in Model 3, as well as the remaining 11 factors, in order to explore the independent associations of each individual factor with CVD when adjusted for a combination of other factors. This adjustment provides a more precise estimate of the independent risk of a single factor with CVD. It was found that multiple factors showed significant associations with CVD under both adjustments.

### Mediation analyses of inflammatory factor on associations of AL with CVD

**Figure 3:**
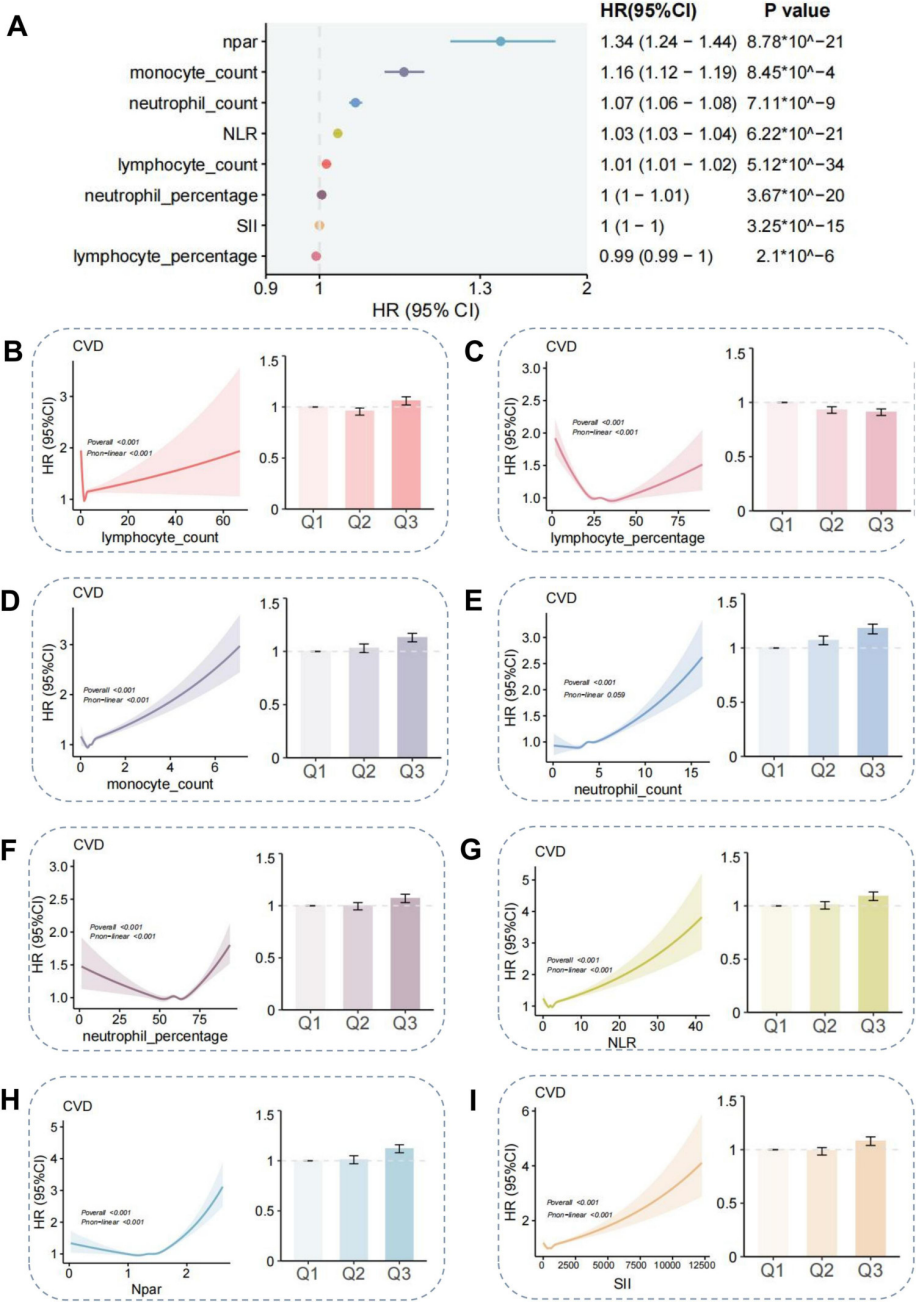
Mediation analysis of inflammatory markers for the AL-CVD association. Panel layout: **(A)** Selection of inflammatory mediators of the AL-CVD association; **(B–I)** indirect effect estimates and 95% CIs for each candidate mediator (neutrophil count, SII, monocyte count, NLR, neutrophil %, lymphocyte %, lymphocyte count, and NPAR), with proportion mediated annotated.

**Figure 4:**
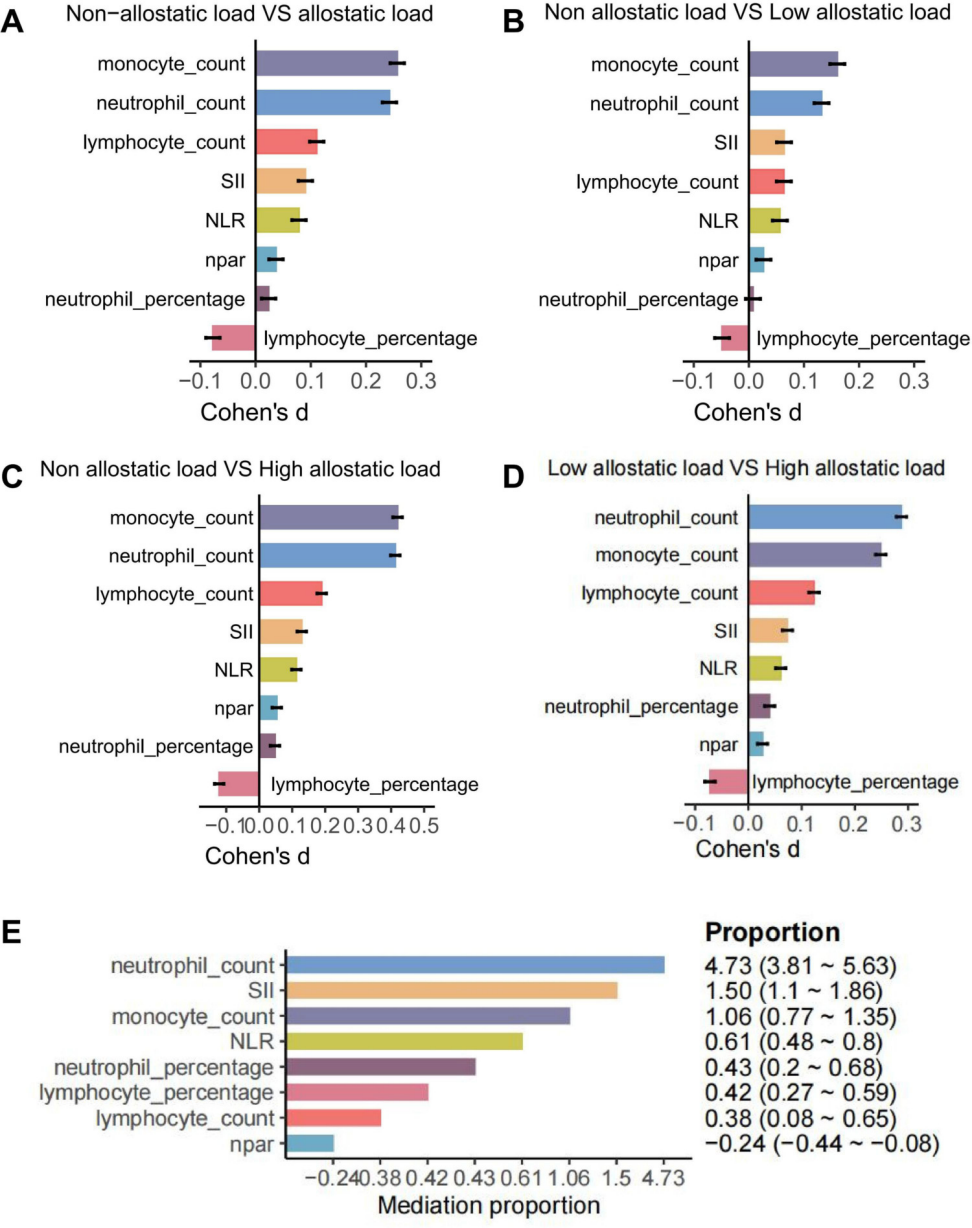
Differences in inflammatory mediators across allostatic load (AL) tertiles and mediation summary. **(A–D)** Cohen's *d* for high vs. low levels of eight mediators across no-AL, low-AL, and high-AL groups. **(E)** Mediation summary showing neutrophil count mediates 4.73% of the AL-CVD association.

As a stress load score, AL has been demonstrated to influence the development of disease by causing oxidative stress and other mechanisms (18). Furthermore, the development of CVD has been shown to be closely related to oxidative stress and other factors (19). Therefore, it can be hypothesised that AL may act on the development of CVD through inflammatory factor mediation. Initially, an analysis was conducted of the common mediating inflammatory factors, and a screening was performed for inflammatory factors that have an impact on the development of CVD. The inflammatory factors that were initially screened included lymphocyte count, monocyte count, neutrophil count, lymphocyte percentage, monocyte percentage, neutrophil percentage, C-reactive protein, neutrophil-lymphocyte ratio (NLR), lymphocyte-monocyte ratio (LMR), platelet-lymphocyte ratio (PLR), systemic immune-inflammation index (SII) and neutrophil-lymphocyte ratio (Npar). (Supplementary Table S4), from which eight potential mediators were identified (Figure 3A). The mediator analyses of the eight factors screened for their effects on the development of CVD are shown in Figures 3B–I. The three groups were stratified by AL level tertiles and compared based on high and low levels of the eight potential mediators. Cohen's *d* values were subsequently calculated to assess effect sizes(Figures 4A–D). The Cohen's *d* effect size for the no-AL vs. high-AL group was slightly larger than that of the other groups, with some variables approaching 0.5. This suggests that a high AL state may be associated with significant changes in inflammatory markers. These findings indicate that an increased AL burden could contribute to alterations in inflammation and immune function, particularly in Monocyte_count and Neutrophil_count. As illustrated in Figure 4E, a synthetic analysis of the eight mediators of AL-induced CVD development reveals that 4.73% of the associations between AL and CVD may be mediated by neutrophil_count.

## Discussion

To investigated that whether AL is a relevant indicator of CVD, several Cox regression models based on the UK Biobank database were developed, AL was positively associated with CVD risk after adjusting for all covariates included in our study, with AL scores of 1, 2, 3, 4, 5, and > = 6 had hazard ratios(95% CI) of 1.23(1.14–1.33), 1.46(1.36–1.58), 1.57(1.46–1.69), 1.71(1.59–1.85), 1.88(1.74–2.03), and 2.15(1.99–2.33). In subgroup analyses, multiple factors demonstrate HRs greater than 1 and CIs that are narrow and do not span 1, suggesting that these factors significantly increase the risk of CVD. Restricted cubic spline analysis showed a non-linear correlation between AL and the occurrence of CVD. The Cohen's d effect size between the no AL vs. high AL groups suggests that a high AL state may be associated with significant changes in inflammation-related markers. Neutrophil_count, SII, Monocyte_count, NLR, Neutrophil percentage, Lymphocyte_percentage, lymphocyte_count and Npar were identified as mediator between AL and CVD and 4.73% of that association was likely to be mediated by Neutrophil_count. Although neutrophil count mediated 4.73% of the AL–CVD association, even modest indirect effects can be meaningful at population scale. Neutrophils and neutrophil extracellular traps (NETs) contribute to endothelial dysfunction, plaque progression, and thrombosis, consistent with our findings (20, 21). However, our mediation analysis is limited by single-time-point biomarker assessment and potential temporal ambiguity between exposure, mediator, and outcome; thus, the proportion mediated should be interpreted cautiously.

Stress is commonly categorized in the literature into 3 broad categories: epidemiologic, psychological, and biological stress (22). Chronic stress biology may accelerate atherosclerosis through sustained neuroendocrine activation that skews leukocyte trafficking and primes innate immunity, increases oxidative stress, and impairs endothelial function (23). Contemporary vascular immunology work places inflammation at the core of atherogenesis, with evidence that neutrophil-dominated responses and NETs promote plaque growth and thrombosis (24–26). Our results align with this paradigm, indicating that higher AL—an index of multisystem dysregulation—is associated with greater incident CVD, with a measurable neutrophil-related component (27). AL, as a composite indicator, integrates multiple stress-related biomarkers to assess the cumulative impact of chronic stress on health. By reflecting the dysregulation of physiological systems, AL helps evaluate the long-term effects of stress on disease risk.

Extensive investigations by McEwen and colleagues have elucidated the pivotal role of chronic stress in disease pathogenesis through persistent activation of key neuroendocrine pathways, particularly the hypothalamic-pituitary-adrenal (HPA) axis and the sympathetic-adrenal-medullary (SAM) system (28, 29). Prolonged stimulation of these stress-responsive systems induces a chronic pro-inflammatory milieu characterized by elevated circulating levels of pro-inflammatory cytokines such as IL-6, TNF-α, and CRP. This sustained inflammatory state disrupts the delicate homeostatic balance of endothelial-derived vasoactive substances, including nitric oxide and endothelin-1, leading to a shift toward vasoconstrictor predominance and impaired vascular reactivity (30, 31). Moreover, excessive oxidative stress and aberrant activation of redox-sensitive transcription factors, notably nuclear factor kappa-light-chain-enhancer of activated B cells (NF-κB), further exacerbate endothelial dysfunction and promote the progression of atherosclerosis (32–34). Dysregulation of these central molecular mediators is accompanied by heightened sympathetic tone and autonomic imbalance, which are increasingly recognized as pathophysiological hallmarks of cardiovascular disease development.

AL, a cumulative measure of physiological wear and tear resulting from chronic stress exposure, has emerged as a reliable preclinical biomarker predictive of both all-cause and cardiovascular-specific mortality. The clinical and public health implications of AL are considerable, as early identification of individuals with high AL may facilitate timely interventions to mitigate adverse outcomes. In oncology, elevated AL is also associated with poorer prognoses. For instance, patients with non-small cell lung cancer or breast cancer exhibiting high AL demonstrate significantly reduced overall survival and higher cancer-specific mortality (35–37).

AL was measured at baseline only; trajectories and cumulative duration of high AL were unavailable, which may attenuate associations toward the null. Second, exclusions for missing covariates and AL components (≈60% of the recruited cohort) may introduce selection bias. Third, because the UK Biobank comprises volunteers who are typically healthier and more health-conscious than the general population—and may differ from other settings in geography, socioeconomic context, recruitment, and health behaviors—external validity may be limited even if internal associations are generally preserved; external validation is warranted. Fourth, as an observational study, residual and unmeasured confounding cannot be excluded despite extensive adjustment. Finally, self-reported lifestyle covariates (dietary intake, physical activity, alcohol use, and smoking) are subject to recall and social desirability biases, whereas biomarker measurements may incur laboratory and temporal variability.

In summary, stress (represented by higher AL) has been adversely associated with a variety of chronic disease-related health outcomes. And in our study, we investigated the relationship between AL and CVD and found that high AL increases the incidence of CVD, which provides some ideas for the prevention and management of CVD patients. However, this finding needs more evidence to confirm and explore the intrinsic link between AL and CVD. In addition to this, there is still some confounding bias in our study, although we have included as many covariates as possible. In addition, recall bias was also present as AL was collected based on self-reported dietary intake over the past 24 h.

## Conclusions

A positive correlation was observed between AL and the risk of CVD, which might be mediated by Neutrophil_count, SII, Monocyte_count, NLR, Neutrophil percentage, Lymphocyte_percentage, lymphocyte_count and Npar.

## Data Availability

The data analyzed in this study is subject to the following licenses/restrictions: Access to the UK Biobank data is restricted to approved researchers under application and data-use agreements (Application ID required) and cannot be publicly shared due to participant confidentiality. Requests to access these datasets should be directed to UK Biobank data are available to bona fide researchers through application to the UK Biobank Access Management System (https://www.ukbiobank.ac.uk/enable-your-research/apply-for-access).

## Supplementary material

Supplementary Table S1 Percentile cutoffs used to construct the allostatic load (AL) score. 25th/75th percentile thresholds for the 12 biomarkers at baseline, indicating risk scoring direction (≥75th considered high-risk for most markers; ≤25th for HDL-C and IGF-1).

Supplementary Table S2 Association between AL (per 1-unit increase) and incident CVD. Cox proportional hazards models reporting hazard ratios (HRs) and 95% CIs per 1-unit increase in AL across sequential adjustments (Model 1-3). Model covariates listed in Methods.

Supplementary Table S3 Incident CVD risk by discrete AL scores or categories. Adjusted HRs (95% CIs) for incident CVD comparing AL = 1, 2, 3, 4, 5, and ≥6 versus AL = 0 (reference), and/or AL categories (e.g., none, low, high). Includes P for trend and nonlinearity where applicable.

Supplementary Table S4 Initial screening of inflammatory indices and mediator selection. Twelve candidate inflammatory indices (lymphocyte, monocyte, neutrophil counts/percentages; CRP; NLR; LMR; PLR; SII; NPAR) with screening metrics and retention status for mediation analyses.
