# Supplementary figures and images for: Allostatic load-cardiovascular disease associations and the mediating effect of inflammatory factors: a prospective cohort study
Source: Front Cardiovasc Med. 2026 Jan 5;12:1724572. doi: 10.3389/fcvm.2025.1724572 (PMC12813168; doi:10.3389/fcvm.2025.1724572)

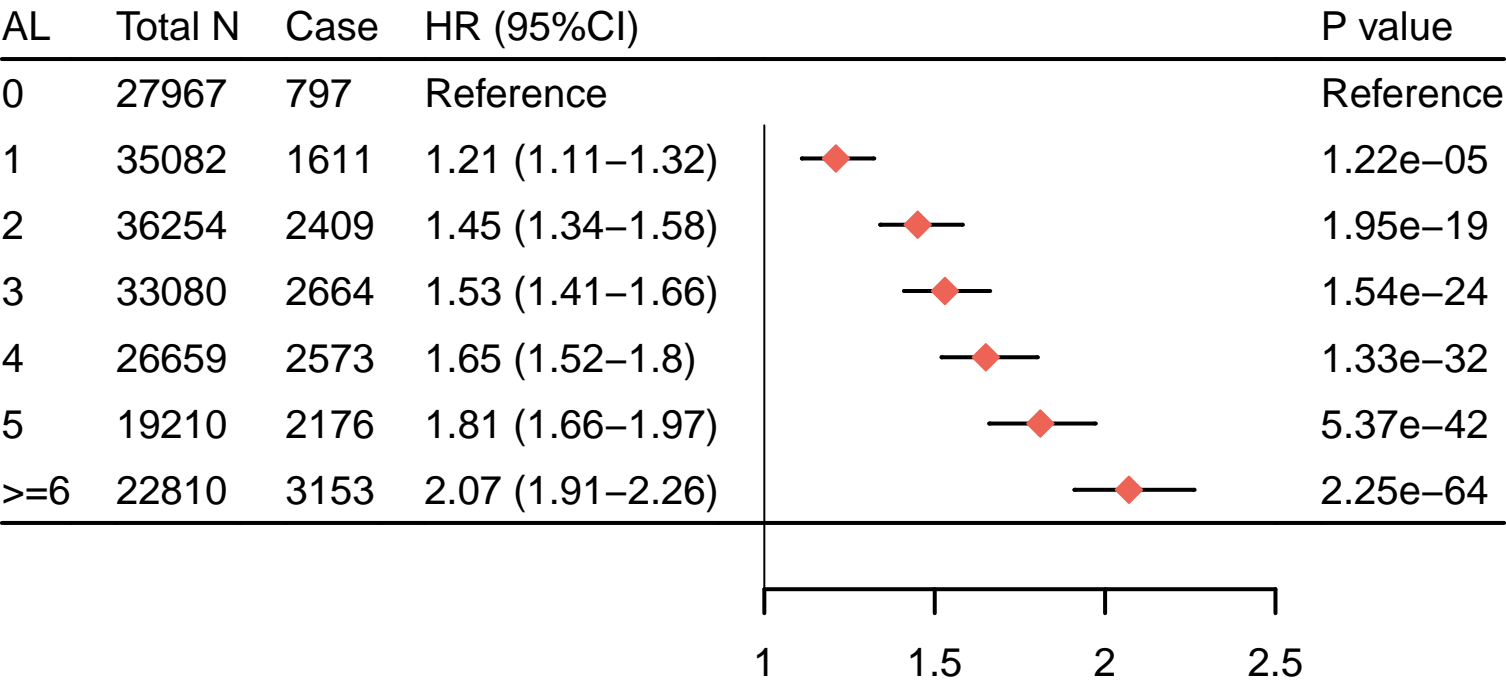

Supplement: Supplementary file 5 [file Image1.pdf]

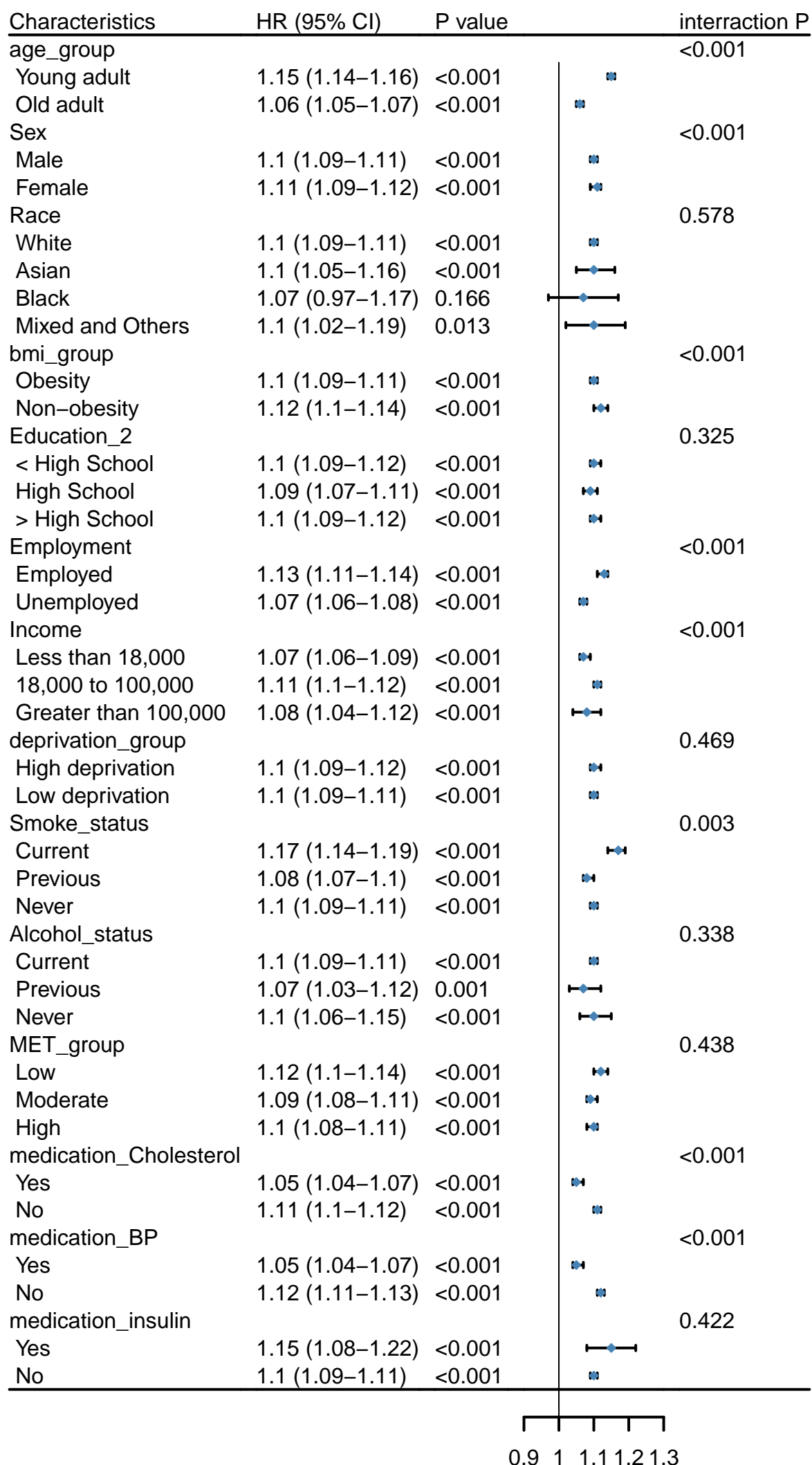

Supplement: Supplementary file 6 [file Image2.pdf]
